# A neglected neurodegenerative disease: Adult-onset globoid cell leukodystrophy

**DOI:** 10.3389/fnins.2022.998275

**Published:** 2022-09-07

**Authors:** Guode Wu, Zhenhua Li, Jing Li, Xin Li, Manxia Wang, Jing Zhang, Guangyao Liu, Pengfei Zhang

**Affiliations:** ^1^Department of Neurology, Lanzhou University Second Hospital, Lanzhou, China; ^2^Department of Magnetic Resonance, Lanzhou University Second Hospital, Lanzhou, China

**Keywords:** globoid cell leukodystrophy, Krabbe disease, adult-onset GLD, galactosylceramidase, GALC, psychosine

## Abstract

Globoid cell leukodystrophy (GLD), or Krabbe disease (KD) is a rare neurodegenerative disease, and adult-onset GLD is more even neglected by clinicians. This review provides detailed discussions of the serum enzymes, genes, clinical manifestations, neuroimaging features, and therapies of GLD, with particular emphasis on the characteristics of adult-onset GLD, in an attempt to provide clinicians with in-depth insights into this disease.

## Introduction

Globoid cell leukodystrophy (GLD), or Krabbe disease (KD), is a rare neurodegenerative disorder of the nervous system caused by autosomal-recessive lysosomal dysfunction. Specifically, the mutation in the gene encoding galactosylceramidase (GALC) leads to the deficiency of β-galactosidase activity, which induces the accumulation of neurotoxic galactosylsphingosine [psychosine (PSY)] in oligodendrocytes and Schwann cells, thereby eventually causing progressive demyelination and secondary axonal degeneration in the central (CNS) and peripheral nervous systems (PNS) (Graziano and Cardile, 2015). According to the age of onset, GLD can be clinically classified into early infantile onset, late infantile onset, late childhood onset, adolescent onset, and adult onset, with early infantile phenotype accounting for about 90% of GLD (Duffner et al., 2009a,^2012^), while late adult onset GLD is rarely reported. Thus, clinicians are less diagnostically sensitive to adult phenotype, especially the late adult onset, which causes frequent misdiagnoses and mistreatment.

## History of research on globoid cell leukodystrophy pathogenesis

### There are three milestones in the research history of globoid cell leukodystrophy

The first milestone includes the early 19th century, when the Danish neurologist Knud Haraldsen Krabbe first described the pathological features of five infant patients with injured white matter and cerebellum as “diffuse sclerosis of the brain”. In 1924, Collier (Wenger et al., 2016) provided a full description of the globular swelling of multinucleated bodies (multinucleated macrophages) and astrocytic hyperplasia in infant patients, which may be the histopathological features of this disease.

Second, in the early 1970s, Malone and Suzuki demonstrated an extremely low or lost activity for a lysosomal enzyme used for degrading galactosylceramide (GalCer) in GLD, the kind of lysosomal enzyme known as galactocerebrosidase or galactosylceramide β-galactosidase (GALC). In the settings of the lack of GALC, PSY, with a neurotoxic effect, would accumulate in macrophages and nerve cells, especially oligodendrocytes and Schwann cells, eventually causing hypomyelination, known as the “Psychosine hypothesis” (Suzuki and Suzuki, 1970). Therefore, enzymatic determination of GALC activity is a fundamental method for the early diagnosis and screening of GLD in newborns worldwide, especially in Europe and the United States (Macarov et al., 2011; Schrier Vergano et al., 2022).

Third, in the 1990s, Chen (Chen et al., 1993) first cloned human GALC complementary DNA (cDNA) from the urine of a GLD patient. Specifically, the entire length of GALC cDNA consisted of 3,795 bp, the coding region was 2,007 bp in size, the 5’ untranslated sequence was 47 bp in size, and the 3’ untranslated sequence was 1,741 bp in size. Thus, the researcher mapped the gene to human chromosome 14 by an *in situ* hybridization with a cDNA probe, specifically chromosome 14q13 region (Cannizzaro et al., 1994).

## Research on *GALC* genes and protein

Undoubtedly, significant efforts have been made throughout the research history of GLD because it is challenging to obtain protein sequencing with sufficient purity from human tissues, including the liver, brain, and even placenta. In the past two decades, a small amount of pure GALC has been purified from urine (Chen and Wenger, 1993). After the amino acid sequence has been obtained, human GALC cDNA has been successfully revealed (Luzi et al., 1995), consisting of 17 exons and 16 introns. Additionally, compared with lesser exons, the introns range in size from 247 bp for intron 2 to about 12 kb for intron 10. Based on the above-mentioned studies, Cannizzaro (Cannizzaro et al., 1994) confirmed that *GALC* gene is located on human chromosome 14 by linkage analysis, and mapped it to chromosome 14q13 region by an *in situ* hybridization with a cDNA probe. The *GALC* gene may encode the precursor protein of GALC, which was processed into a mature form by being cut into 50-kDa N-terminal fragments and 30-kDa C-terminal fragments by proteases in lysosomes (Nagano et al., 1998). Deane (Deane et al., 2011) has discovered the crystal structures of GALC enzyme and revealed three distinctive domain architectures contributing to substrate binding. Besides, some researchers cloned GALC cDNA from other species, including dogs (Victoria et al., 1996), mice (Sakai et al., 1996), and rhesus monkeys (Luzi et al., 1997), which laid a foundation for establishing animal models of GLD for the future.

**FIGURE 1:**
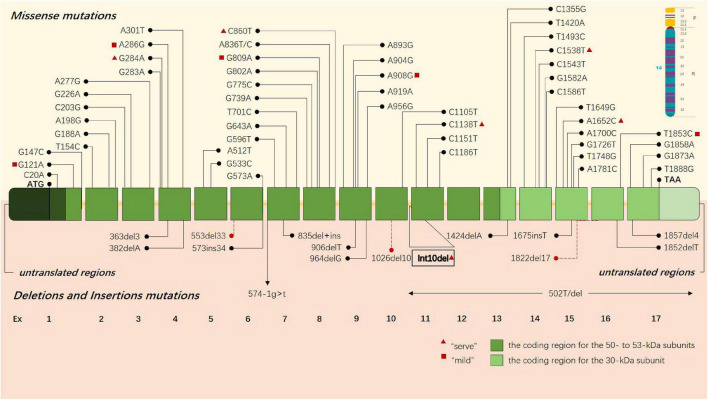
GLD-causing common sites and types of gene mutations are presented in the schematic drawing of GALC gene. Missense mutations are above the gene, and deletions and insertions are shown below the gene. Each box represents an exon, the dark green boxes contain the coding region for the 50- to 53-kDa subunits, and the light green boxes contain the coding region for the 30-kDa subunit. The red triangle indicates that this gene may cause a severe clinical phenotype, and the red square indicates that the gene may lead to a mild clinical phenotype.

Early studies preliminarily revealed that the deletion of 30 kb subunits encoding non-functional proteins is the most common pathogenic mutation of GLD, accounting for about 35%–50% of cases (Rafi et al., 1995). This deletion occurs in the C > T transition polymorphism at position 502 of cDNA, inducing the elimination of the coding regions of seven exons, including all 30-kDa subunits and about 15% of 50-kDa subunits. An increasing number of pathogenic gene mutations has been reported in the past three decades (Wenger et al., 2021). Until now, a total of 296 *GALC* gene mutations related to GLD have been cataloged in the Human Gene Mutation Database (HGMD), including missense mutations, non-sense mutations, deletion, and insertion (Figure 1), and the sites and types of gene mutations have regional and racial variation (Rafi et al., 1996; Kleijer et al., 1997; Xu et al., 2006; Zayed, 2015). Unlike infantile GLD with mutations in the central domain of the coding region, adult GLD has GALC mutations at the N or C terminals, and most mutations are located in GALC gene region encoding 50-kDa subunits (Furuya et al., 1997; Wenger et al., 1997). There is a certain correlation between the clinical manifestations and genotypes of GLD in terms of theory: the type and location of gene mutations would affect GALC protein and its activity; hence, it is ideal to be able to classify a disease-causing variant as “severe” or “mild” to predict as the possible type and severity of GLD a patient will have. For instance, c.857G > A (809G > A) mutation and 30-kb deletion are more common in adult onset (De Gasperi et al., 1996), and p.G286D is common in patients with late childhood GLD (De Gasperi et al., 1999; Tappino et al., 2010). c.169G > A (121G > A) mutation is associated with the late-infantile KD form, especially in Japan (Hossain et al., 2014). and Furthermore, the activity of residual β-galactosidase can be detected in these patients. The exploration into the correlation between the clinical manifestations and genotypes of GLD contributes to predicting patients with GLD during screening, but high variability of polymorphic changes on allelic genes complicates the relationship between genotype and clinical phenotype.

We summarized some pathogenic heterozygous mutations associated with adult-onset GLD by gathering and analyzing previously published literature (Table 1).

**TABLE 1:** The pathogenic genes associated with adult onset GLD.

Gene genotype (Heterozygous mutations)	Age of onset	Comments
**p.T96A**; (c.286A > G) **p.D171V**; (c.512A > T) (Luzi et al., 1996)	45	Mild
**G286D**; (c.809G > A)**K343AfsX3**; (1027_1036delAAGACAGTTG) (De Stefano et al., 2000)	42	Mild
p.T633Tfs*2; (c.1899delG) **p.T529M**; (c.1586C > T) (Zhong et al., 2020)	27	Severe
**p.L634S**; (c.1901T > C) **p.L95fs**; (c.283_284del) (Zhang et al., 2021)	22	Mild
**30kbdel**; (IVS10del30kb) **p.G286D**; (c.809G > A) (Harzer et al., 2002)	50	Mild
**p.G496S**; (c.1486G > A) **p.G569S**; (c.1705G > A) (Tokushige et al., 2013)	60	Mild
**30kbdel**; (IVS10del30kb) **G622S**; (c.1864G > A) (Debs et al., 2013)	24	Mild
**p.V681M**; (c.2041G > A) **c.1911 + 1_1911 + 5delGTAAG** (Yang et al., 2013)	29	Mild
**p.G43R**; (c.127G > C) **p.I66M** (c.198A > G) + I289V (Lim et al., 2016)	35	Mild
**p.N228_S232delTP** (c683_694delinsCTC) **p.G286D**; (c.857G > A) (Hossain et al., 2014)	30	Mild
**p.L634S**; (c.1901T > C) **p.L634X**; (c.1901delT) (Zhang et al., 2018)	20	Mild
**p.L634S** (c.1901T > C) **p.Y335X**; (c.1005C > G) (Meng et al., 2020)	40	Mild
**p.G286D**; (c.857G > A) **p.Y490N**; (c1468T > A) (Iacono et al., 2022)	45	Mild

## Pathogenesis

### Normal physiological metabolism of galactocerebrosides and psychosine

**FIGURE 2:**
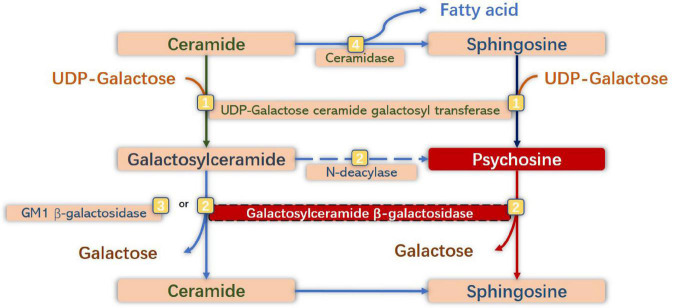
Anabolic and catabolic pathways of galactosylsphingosine [psychosine (PSY)] and galactosylceramide. Galactosylceramide is one of the significant glycosphingolipids of oligodendrocytes (OLs) and Schwann cells, which is synthesized by galactosylation of ceramide by UDP-galactose ceramide galactosyl transferase (UDP-galactose CGT) (Graziano and Cardile, 2015), and is degraded to ceramide by degalactosylation by both galactosylceramide β-galactosidase (GALC) (Duffner et al., 2009a) and GM1 β-galactosidase (Duffner et al., 2012). Sphingosine is synthesized by hydrolysis of ceramide by ceramidase (Wenger et al., 2016). PSY is synthesized by galactosylation of sphingosine by UDP-galactose CGT, and from galactosylceramide by *N-*deacylase (Suzuki and Suzuki, 1970), and degraded to sphingosine by degalactosylation by GALC. Under GALC-deficient conditions, neurotoxic PSY accumulates to high levels in macrophages (globoid cells) and neural cells, especially in OLs and Schwann cells.

Normally, PSY is a cytotoxic lysolipid that can be generated either by the galactosylation of sphingosine by UDP-galactose ceramide galactosyl transferase (CGT) (UDP-galactose-CGT), or by the deacylation of GalCer by *N-*deacylase (Lgisu, 1989; Kanazawa et al., 2000). Having a critical role in the normal physiological mechanism, UDP-galactose-CGT in oligodendrocytes and Schwann cells participates in the synthesis of myelin lipids and myelin formation (Schulte and Stoffel, 1993; Marcus et al., 2006) and plays a vital role in maintaining the function and structure of membranes of other cells (Dupree et al., 1999). The expression of CGT is high in oligodendrocytes and Schwann cells, but lower in neurons and astrocytes (Schaeren-Wiemers et al., 1995; Pernber et al., 2002). Under In GALC-deficient conditions, PSY would accumulate to high levels in tissues, especially in the brain (Suzuki, 1998). Notably, the primary substrate of GALC is hydrolyzed by GM1 ganglioside β-galactosidase in this situation. Therefore, the level of GalCer in tissues would not be high. Additionally, the number of cells expressing UDP-galactose-CGT may be reduced due to the susceptibility to the toxic effect of PSY, which would further increase the accumulation of PSY (Nagara et al., 1986; Ida et al., 1990; Figure 2).

### The correlation between the myelination stage and globoid cell leukodystrophy

The correlation between the myelination stage and the onset of diseases demonstrates that GLD-induced myelin sheath injuries often occur at the most active myelination stage. The spinal cord is one of the first CNS regions to obtain myelin, and myelination occurs from the gestational age of 20 weeks to 2 years in the spinal cord (Tanaka et al., 1995). Myelination in the pyramidal tract occurs from the gestational age of 25 weeks to 2–3 years (Kinney et al., 1988). Myelination in the posterior corpus callosum occurs from 3 to 8 months after birth to adolescence and adulthood (Barkovich et al., 1988; Courchesne et al., 2000). Myelination in the parieto-occipital white matter begins from at 4–6 months after delivery and reaches 50% at 11–14 months after birth (Bartzokis et al., 2001; Deoni et al., 2011). The most significant changes in myelination occur between midgestation and the end of the second postnatal year. The temporal and spatial pattern of myelination seems to explain the temporal features of myelin sheath abnormalities in GLD. According to studies based on magnetic resonance imaging (MRI), spinal cord involvement precedes intracranial abnormalities in infantile GLD (Vasconcellos and Smith, 1998). In infantile GLD (within 2 years of birth), MR signal abnormalities appear at the active myelination stage, including corticospinal projection fibers (pyramidal tract), posterior corpus callosum, and parietal white matter. Besides, it can also be used to explain that the abnormalities of the cerebellar white matter are only observed in early GLD (including early infantile and late infantile types) but not in adult GLD. We suggest that the myelination of cerebellar white matter begins during pregnancy and reaches the adult level at the age of 3–18 months. The myelination of the cerebellar white matter reaches the adult level before the onset of adult GLD. Hence, adult GLD will not be accompanied by cerebellar lesions or related clinical signs, especially in patients with late adult GLD.

### Mechanism of psychosine toxicity in the central and peripheral nervous systems

Psychosine is a metabolic by-product of the precursor of GALC causing membrane structure changes due to its detergent-like properties, inducing oligodendrocyte death and myelin sheath loss. However, it has been proved that PSY could induce abnormal membrane structure by changing the structure and function of lipid rafts in animal experiments (White et al., 2011; Hawkins-Salsbury et al., 2013). As has been revealed in many studies, the mechanisms of PSY toxicity in the CNS and PNS also include PSY-induced stress and proapoptosis signaling pathways, PSY-induced immune and inflammatory responses, PSY-induced mitochondrial energy disorder, peripheral nerve injury, and cerebral microvascular dysfunction (neurovascular unit damage) (Won et al., 2016). Multiple signaling pathways are involved in oxidative stress (OS) and apoptosis of oligodendrocytes induced by PSY (Jatana et al., 2002; Hamanoue et al., 2004; Pannuzzo et al., 2010). PSY plays a vital role in the apoptosis of oligodendrocytes by activating stress-activated protein kinases (SAPKs) (Jurewicz et al., 2006). In addition, it can induce the activity and expression of cell death signals and simultaneously inhibit cell survival signals, such as phosphoinositide 3-kinase (PI3K) (Arai and Lo, 2010; Won et al., 2013; Graziano et al., 2016). Multinucleated activated microglia or macrophages can be observed in the brain of patients with GLD and animal models from previous studies, especially the activation and proliferation of astrocytes and the proliferation of glial fibrillary acidic protein (GFAP) in the demyelination area of the white matter, implying that immune and inflammatory reactions mediated by microglia and peripheral immune cells are involved in GLD pathogenesis (Kobayashi et al., 1986; Ransohoff and Brown, 2012). Some researchers have thought that the immune and inflammatory responses of GLD are secondary performance. However, it has been confirmed that PSY-induced infiltration of immune cells and overexpression of cytokines, such as tumor necrosis factor-α, interleukins, and chemokine, can further activate transcription factors, making the up-regulation of inflammatory response appear at an early stage of this disease (Wu et al., 2001; Borda et al., 2008; Ripoll et al., 2011; Hawkins-Salsbury et al., 2012; SSnook et al., 2014). The accumulation of PSY would induce mitochondrial dysfunction and adenosine triphosphate (ATP) consumption (Haq et al., 2003; Giri et al., 2006; Voccoli et al., 2014). Currently, the increased activity of secretory phospholipase A2 (sPLA2) induced by PSY is considered the critical pathological mechanism, and its products can inhibit mitochondrial enzyme activity and destroy mitochondrial integrity (Kalous et al., 1992; Hollie et al., 2014; Wu et al., 2021). AMP-activated protein kinases (AMPKs) can be activated during energy deficiency to produce and preserve ATP (Hardie, 2004; Carling, 2005). The accumulation of PSY down-regulates or even inactivates the activity of AMPKs, accelerating the consumption of ATP in oligodendrocytes, causes mitochondrial dysfunction in oligodendrocytes and their inactivation, which has been confirmed to be irreversible (Giri et al., 2008). According to previous studies, the excessive accumulation of PSY can also inhibit the function of peroxisomes, which would disturb metabolism and energy homeostasis (Khan et al., 2005; Haq et al., 2006). Lower motor neuron disease induced by peripheral nerve demyelination is the most common non-central nervous system disorder in GLD, such as peripheral neuropathy and muscle diseases. As has been revealed in current studies, the toxic effects of PSY might directly mediate injuries in neuron, axon, and neuromuscular junctions (Castelvetri et al., 2011). PSY induces the production of α-synuclein, a neuronal inclusion, leading to abnormalities of neurofilaments by relieving the regulation of PP1 and PP2A phosphatases (Cantuti-Castelvetri et al., 2012) and inducing an axon transport imbalance by activating glycogen synthase kinase 3β (GSK3β) (Cantuti Castelvetri et al., 2013). This results in axon damage and neuron death in the PNS. PSY can reduce peripheral nerve conduction by activating caspase and inhibiting Akt activities, aggravating the damage to neuromuscular junctions.

In this article, we propose that GLD can involve neurovascular units, which may be the ultimate pathological mechanism contributing to the commonalities of CNS degeneration. Abnormalities of microvasculature and disruption of the blood-brain barrier (BBB) in patients with GLD, mainly including perivascular space enlargement, cerebrovascular permeability increases, endothelial morphology changes, macrophage infiltration, and microvascular structure changes, would develop (Giacomini et al., 2015; Belleri and Presta, 2016). It has been confirmed in some studies that the decreased proliferation and migration of endothelial cells induced by PSY and the declined response to angiogenic factors may hinder angiogenesis. The destroyed actin cytoskeleton and the down-regulated expression of tight junction proteins induced by PSY might lead to changes in microvascular ultrastructure and permeability (Belleri et al., 2013). The damage of PSY to oligodendrocytes would cause secondary damage to astrocytes and neurons, accompanied by microvascular injury (Cantuti Castelvetri et al., 2015).

## Clinical features

According to the age of onset, GLD can be clinically classified into five types: early infantile type (onset at 0–6 months), late infantile type (onset at 7–12 months), late childhood type (onset at 13 months-10 years), adolescent type (onset at 11–20 years), and adult type (onset at > 21 years) (Hagberg, 1984). There is a close correlation between clinical symptoms and the age of onset in GLD patients. Patients with the early infantile type account for about 85–95% of cases, and the early infantile type has the worst prognosis, and the general median survival time is 8–36 months (Duffner et al., 2011). The clinical course is usually divided into three clinical stages: non-specific symptom stage, symptom progression stage, and end burnt-out stage. At the non-specific symptom stage, infants often present with crying, irritability, poor feeding, vomiting, and myoclonic seizures (Duffner et al., 2009b), accompanied by peripheral neuropathy (Siddiqi et al., 2006). At the symptom progression stage, infants often present with optic atrophy, aggravated epileptic seizures, and the rapid progression of psychomotor degeneration. At the end burnt-out stage, infants completely lose autonomous movement, require nasal feeding, and eventually die of infection and respiratory failure (Chabali et al., 1997). The late infantile type is characterized by progressive deterioration of motor function, spastic paraplegia, cerebellar ataxia, hemiplegia (Fiumara et al., 2011). About 12.5% of patients with late infantile GLD have a visual impairment or even blindness (Lyon et al., 1991). Compared with the early-infantile onset, the late-infantile onset type has a significantly extended lifetime of 2∼14 years (Barone et al., 1996). The late childhood type is a transitional clinical phenotype from late infantile type to adolescent type. The symptoms related to motor functions of the adolescent type are not as significant as those in the early infantile or late infantile types. However, spastic paralysis or cerebellar ataxia can still occur in the adolescent type, and visual dysfunction is the most common clinical symptom in the adolescent type (Phelps et al., 1991). With aging, symptoms would become alleviated in these patients (Arvidsson et al., 1995).

The adult type has the lowest incidence, with chronic progressive pyramidal tract injuries as the main manifestation. Additionally, patients with this type present with chronic progressive spastic paralysis and gait disorders, with or without peripheral nerve injuries, mental disorders, cognitive disorders, bulbar paralysis, and tremor (Henderson et al., 2003; Tokushige et al., 2013). Almost all patients with adult GLD present with chronic progressive spastic paraplegia or walking difficulties, and sometimes the symptoms may be mild (Paiva et al., 2022), but a patient can also present with acute hemiparesis (Mamada et al., 2016). Most patients also develop peripheral nerve injuries (Sabatelli et al., 2002), and isolated peripheral nerve injury occur in some patients (Marks et al., 1997; Adachi et al., 2016). In these patients, asymmetric peripheral nerve conduction abnormalities have been revealed in some electrophysiological studies. Specifically, prolonged F wave incubation period, reduced motor nerve conduction velocity homogeneity, and prolonged distal incubation period are the most common abnormal manifestations, which are consistent with peripheral nerve demyelination (Matsumoto et al., 1996). When the patient is complicated by pes cavus, differential diagnosis with respect to complicated forms of Charcot-Marie-Tooth disease or hereditary spastic paraplegia is necessary (Malandrini et al., 2013). However, a study conducted by Adachi has demonstrates demonstrated that there are many obvious thin thin-myelinated nerve fibers in sural nerves of patients with GLD, and the density of myelinated nerve fibers decreases (Madsen et al., 2019). However, there is no characteristic “onion ball” structure of peronial peroneal myoatrophy, which may be one of the key points for the differential diagnosis between demyelination and dysmyelination. Mild cognitive impairment (MCI) and psychiatric symptoms often occur in patients with adult GLD (Xia et al., 2020). Compared with adolescent GLD, optic atrophy is less likely to occur in adult GLD (Madsen et al., 2019). Notably, cerebellar ataxia would not occur in patients with adult GLD, and this temporal and spatial pattern correlates with the pathological mechanism of this disease. The majority of patients have slow or insidious progress of dyskinesia, and sometimes cognitive impairment, but such patients have survived up to 10–15 years after the initial onset of symptoms, according to the literature (Liao et al., 2014).

It is difficult to evaluate the clinical manifestations of adult GLD in real-world clinical practice, it is impossible to assess whether these patients are completely normal before the age of 20 years or have some mild clinical symptoms that are difficult to observe, hindering the further exploration into adult GLD.

## Imaging features

### Features of white matter lesions

Bilateral symmetrical white matter lesions are a mark of most hereditary white matter lesions and a few secondary white matter lesions. Patchy white matter lesions are common in inflammatory demyelination of the CNS, such as multiple sclerosis, and scattered multiple punctate lesions are common in ischemic white matter lesions (Schiffmann and van der Knaap, 2009). MRI features of the brain have been correlated with the age of onset of GLD patients (Abdelhalim et al., 2014; Muthusamy et al., 2019; Andriescu et al., 2020). For example, white matter hyperintensities (WMHs) mainly involve the cerebellar white matter and the dentate nucleus at the early and late stages of infantile GLD. The cerebellum of adolescent GLD patients is basically normal, but WMHs involve the parietal white matter.

**FIGURE 3:**
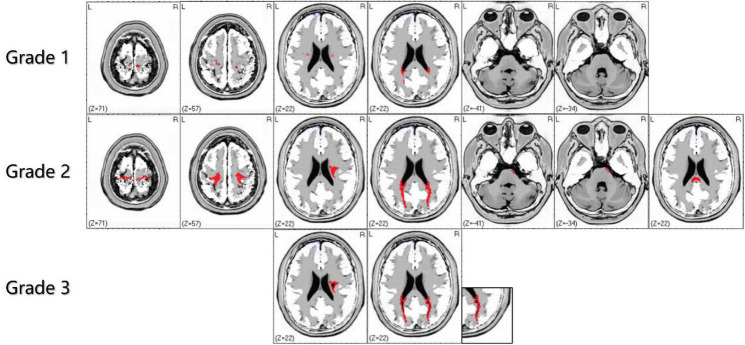
The characteristics of white matter lesions should be graded by imaging, including grade 1: sporadic punctate white matter lesions; grade 2: patchy or continuous linear white matter lesions; grade 3: obvious cavity-like changes in the lesion site. In the supratentorial and infratentorial structures, the damage degree of the corticospinal tract and medial lemniscus should shall be highlighted in the transverse and coronal sections of brain magnetic resonance imaging (MRI). Grade 1 is defined as discontinuous white matter lesions, and grade 2 is defined as continuous white matter lesions. The red area refers to WMHs, and the black part of the red area refers to necrosis or cavity formation in WMHs.

Bilateral WMHs usually occur in adult GLD patients and are limited to the parietal white matter (temporo-parietal junction), corona radiata, optic radiation, proximal corticospinal tract, posterior lateral periventricular white matter, and corpus callosum (Wang et al., 2007; Tokushige et al., 2013), with or without brain and spinal cord atrophy (Bajaj et al., 2002). Noteworthy, cerebellar white matter lesions seldom occur in adult GLD patients. Due to the undefined correlation between gene mutation types and clinical phenotypes, the focus of recent studies has been placed on the classification and scoring of intracranial white matter abnormalities, to identify the correlation of the site and severity of white matter lesions with clinical phenotypes (Cousyn et al., 2019). Based on the analysis and summary of the MRI features of adult GLD patients in our clinical center, we simplified the imaging scoring criteria for GLD to better define the pattern of leukodystrophy and provide treatment opportunities earlier (Figure 3). In the revised scoring criteria, we still highlighted the significance of “continuity of lesions” in the site and severity of white matter lesions, which correlated with the scoring of the lesion site. Discontinuous supratentorial white matter lesions are mainly involved in four parts in the transverse section of brain MRI, including pre- and postcentral gyrus, corona radiate, posterior lateral periventricular white matter, and optic radiations. The degree of white matter lesions of the corpus callosum should be highlighted in the median sagittal section. In theory, the higher the score, the more serious the clinical symptoms. The sensitivity and specificity of the grading scale should still be verified by numerous clinical data.

Adult GLD patients usually have a relatively slow progression of white matter lesions. Therefore, the clinical process from an acute attack to rapid aggravation may be misdiagnosed as acute cerebral infarction or inflammatory demyelination of the CNS, such as multiple sclerosis (Debs et al., 2013; Mamada et al., 2016). Therefore, dynamic and longitudinal image follow-up shall be regularly performed to systematically evaluate the accuracy of diagnosis.

### Other imaging features

Other imaging features include unilateral white matter involvement, advanced brain atrophy with different degrees (Lemmens et al., 2011). Optic neuropathy, optic chiasma lesions, and enhanced oculomotor nerves and trigeminal nerves are common findings in infantile patients (Beslow et al., 2008; Patel et al., 2008; Ganesan et al., 2010; Garcia et al., 2010; Quintas-Neves et al., 2020), which have rarely been reported in adult-onset GLD. Compared with often reported thickened cauda equina nerves in early-infantile and late-infantile Krabbe disease patients (Hwang et al., 2016), brachial plexus enlargement is a more common MRI finding in adult-onset Krabbe disease (Hiyama et al., 2016).

As the study of white matter lesions at an early stage by magnetic resonance spectroscopy (MRS) confirmed, the pattern of metabolic alterations as detected by MRS in infantile-onset, juvenile-onset, and adult-onset has been related to age (Brockmann et al., 2003; Romano et al., 2009), and the concentration of white matter metabolites in adult GLD patients is close to the normal level. Furthermore, the concentration of *N-*acetylaspartate and *N-*acetylaspartylglutamate decreases; while, that of choline-containing compounds, creatine, and phosphocreatine increases. There is a slight change in the concentration of myoinositol. The spectra of the gray matter show a slight decrease in the concentration of all metabolites (Udow et al., 2012). Diffusion tensor imaging (DTI) is the most valuable neuroimaging method for CNS demyelination, assess the severity of white matter damage in infantile GLD (Provenzale et al., 2005; Escolar et al., 2009; Wang et al., 2011; Poretti et al., 2014). It may detect early white matter injury in asymptomatic neonates, predict motor functions and prognosis after stem cell transplantation (Poretti et al., 2016).

## Therapies

Enzyme replacement therapy (ERT) has been emphasized in the preliminary studies on GLD treatment. This therapy has been adopted from the treatment of lysosomal storage diseases (Desnick and Schuchman, 2012). However, it is difficult to achieve efficacy owing to the blocking effect of the BBB, especially clinical symptoms exist at diagnosis (Escolar et al., 2005). Currently, hematopoietic stem cell transplantation (HSCT) is the optimal viable therapy for GLD treatment, and these cells are mainly derived from the bone marrow (BM) or umbilical cord blood (UCB) (Wright et al., 2017). Moreover, virus-mediated gene therapy, anti-inflammatory and antioxidant therapy, oligodendrocyte transplantation, and substrate reduction therapy have been subjected to drug tests based on animal models (Mikulka and Sands, 2016). However, a single therapy cannot achieve satisfactory efficacy. GalCer deficiency in GLD can trigger various pathogenic mechanisms, suggesting that there may be multiple targets during the treatment of this disease. Therefore, the focus of recent studies has been placed on different combined therapies based on these therapeutic regimens. Additionally, treatment strategies emphasize symptomatic and supportive treatment from multidisciplinary collaboration for patients (Escolar et al., 2016).

### Enzyme replacement therapies

Lysosomal storage diseases, such as Fabry disease, are usually treated with ERT (Griffiths, 1989). The core of treatment lies in providing active GALC for dysfunctional cells and tissues in the CNS and PNS. In 2005, Lee (Lee et al., 2005) prolonged the life span of twitcher mice to 47 days by an intraperitoneal injection of recombinant GALC. However, this ERT based on intraperitoneal injection could not provide a long-term effect due to the existence of the BBB. In 2007, Lee (Lee et al., 2007) improved the activity of GALC in the CNS of twitcher mice by injecting recombinant GALC into the ventricle. However, there are two defects in this therapy. First, repeated invasive injections are required to maintain high GALC concentration. Second, multiple injections may be required because GLD widely influences the CNS and PNS. These defects hinder the application of ERTs in clinical practice.

### Hematopoietic stem cell transplantation

Currently, HSCT is the standard therapy for GLD treatment. GALC-positive donor cells from BM or UCB are transplanted to the CNS to continuously provide GALC and reduce the degree of neuroinflammation (Reddy et al., 2011). Bone marrow transplantation (BMT) is widely used in the early treatment of this disease. At present, umbilical cord blood transplantation can be used for the treatment of patients with early infantile GLD. However, early screening should be performed on newborns (Kwon et al., 2018). The process of hematopoietic stem cells performing a therapeutic role is called cross-correction. Functional GALC secreted by hematopoietic stem cells is ingested and combined with mannose receptors on the surface of defective cells in the CNS through mannose-6-phosphate (M6P), and then transported to lysosomes through endocytosis (Neufeld and Fratantoni, 1970). Both the animal experiment results and the evidence of GLD treatment demonstrate the effectiveness of HSCT, especially for infantile GLD patients before the appearance of symptoms (Krivit et al., 1998). Further, HSCT has been reported to exert immune regulation by reducing the degree of neuroinflammation (Caniglia et al., 2002). Notably, patients receiving HSCT are at risk of severe graft-versus-host disease (GVHD), and this therapy can improve clinical symptoms only to a certain extent.

### Virus-mediated gene therapy

It has been confirmed in previous animal experiments that virus-mediated gene therapies mediated by adeno-associated virus (AAV) orienting the CNS can improve enzyme activity and enhance behavior and cognitive function in mouse models with lysosomal storage diseases (Passini et al., 2005, 2006). Adenovirus capsid proteins (AAV1, 5, 9, rh10) are currently widely used in GLD treatment (Asokan et al., 2012), and several types of capsid proteins have presented favorable outcomes. After GALC cDNA is delivered by adenovirus to twitcher mice, GALC activity increases several times in the CNS, and the survival time is significantly prolonged compared with untreated mice, by 40–50% (Rafi et al., 2005). Recently, Rafi et al. treated twitcher mice by intravenously injecting AAV2/rh10-mGALC into the tail vein. The results showed that the survival time of treated mice was significantly longer than that of untreated mice, and the GALC activity in the brain, spinal cord, and sciatic nerve reached or exceeded the recombinant wild-type (WT) level (Rafi et al., 2012, 2015a). This implies that multiple injections are not required in virus-mediated gene therapy, which is different from ERT. The efficacy of virus-mediated gene therapy correlates with the improvement of adenovirus vectors, and this therapy is one of the most promising therapies for treating GLD (Rafi, 2016; Nasir et al., 2021; Hordeaux et al., 2022).

### Anti-inflammatory and antioxidant therapy

PSY-related hyperoxidative stress and inflammatory reactions are also the targets for treating GLD. It has been confirmed through animal experiments that ibudilast, a phosphodiesterase inhibitor, can reduce the degree of tremor and demyelination in twitcher mice, but cannot prolong their life span (Kagitani-Shimono et al., 2005). Luzi (Luzi et al., 2009) used indomethacin, ibuprofen, and minocycline to prolong the life of twi-trs mice (an animal model of GLD). The result has shown that indomethacin could significantly reduce pro-inflammatory cytokines, including interleukin (IL)-6 and tumor necrosis factor (TNF)-α. Paintlia (Paintlia et al., 2015) significantly prolonged the average life span of twitcher mice *via* a diet therapy with a high level of vitamin D, leading to a decreased accumulation velocity of PSY in the body, and a mitigated degree of demyelination.

### Oligodendrocyte transplantation

Oligodendrocytes in the CNS are one of the targets in treating GLD. Hence, the ultimate goal of treatment is to stabilize the normal function of oligodendrocytes and other myelin sheath cells. Kuai (Kuai et al., 2015) injected oligodendrocyte precursor cells differentiated from normal mouse embryonic stem cells into twitcher mice. However, the researchers obtained unsatisfactory results. Both the motor function and life span could not be improved through this therapy.

### Substrate reduction therapy

Substrate reduction therapy has shown some prospects in the treatment of lysosomal storage diseases. Although this therapy can only alleviate clinical severity and prolong the life span, GLD can still not be eliminated (Parenti et al., 2015). Galactose ceramide galactosyl transferase (CGT) is the enzyme directly responsible for synthesizing PSY in GLD. However, there is no effective inhibitor for this enzyme. Thus, it is necessary to identify the target enzyme upstream of PSY synthesis for achieving effective treatment (LeVine et al., 2000). However, many downstream metabolites related to these upstream enzymes may be affected.

### Combined therapies

Currently, there are many synergistic regimens based on HSCT, including BMT + endothelial growth factors (Young et al., 2004), BMT + substrate reduction therapy (LeVine et al., 2000), BMT + enzyme replacement therapy (Qin et al., 2012), BMT + virus-mediated gene therapy (Lin et al., 2007; Galbiati et al., 2009; Rafi et al., 2015b), and BMT + gene therapy therapies + substrate reduction therapy (Hawkins-Salsbury et al., 2015). These synergistic regimens outperform single-drug regimens in multiple aspects, including alleviating pathological demyelination, prolonging the life span of twitcher mice, and improving motor function (Bradbury et al., 2021). However, these synergistic regimens are still in the stage of animal experiments, and a long-term research may be required for the real-world application of these regimens for GLD patients.

Among the treatments for globoid cell leukodystrophy, hematopoietic stem cells are used clinically, especially in infants, except for other treatment options, which are basically in the animal experimental stage. There are currently few reports on the treatment of GLD in adults due to the low incidence in this population and the limitations of clinical therapies. According to some case reports, HSCT may be effective in treating adult-onset GLD patients (Sharp et al., 2013). Moreover, the intravenous injection of gamma globulin has been effective in treating adult-onset GLD patients (Fukazawa et al., 2021). Symptomatic and supportive therapies are still the main clinical regimens for treating adult-onset GLD patients.

## A feasible diagnostic idea and criteria

**FIGURE 4:**
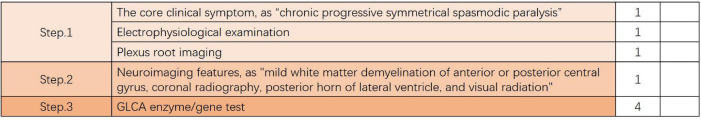
Feasible diagnostic ideas and criteria. If the total score is > 4, it is a confirmed diagnosis; If the total score equals 4 points (without enzyme or gene testing), it is strongly recommended to perform GALC enzyme or genetic testing. If the total score is < 4, differential diagnosis should be focused on, and GALC enzyme or genetic testing should be performed when necessary.

Despite a systematic review of the pathogenesis, clinical manifestations, and neuroimaging features of globoid cell leukodystrophy, the actual clinical diagnosis of this type of CNS degeneration is mains a challenge. Based on our experience, we tried to provide feasible diagnostic ideas and criteria (Figure 4).

Step 1 is to: assess the patient whether they meet the core clinical symptom of the vast majority of adult-onset GLD, which is “chronic progressive symmetrical spasmodic paralysis”, If the conditions are met, one point is added. Further, an electrophysiological examination should be performed to identify whether it is peripheral neuropathy or evaluate whether it is accompanied by peripheral nerve damage; if the conditions are met, one point is added. If the electrophysiological examination results are abnormal, further brachial plexus root imaging is required (it adds the evidence of diagnosis, but is not used as the diagnostic criteria); if the conditions are met, one point is added.

Step 2 is to: assess patient whether the patient meets the imaging core symptoms of most adult-onset globoid cell leukodystrophy are “mild white matter demyelination of anterior or posterior central gyrus, coronal radiography, posterior horn of the lateral ventricle, and visual radiation” (it adds the evidence of diagnosis, but is not used as the diagnostic criteria); if the conditions are met, one point is added.

Step 3: is to determine the affected GLCA enzyme, or perform genetic testing; if the conditions are met, four points are added.

**FIGURE 5:**
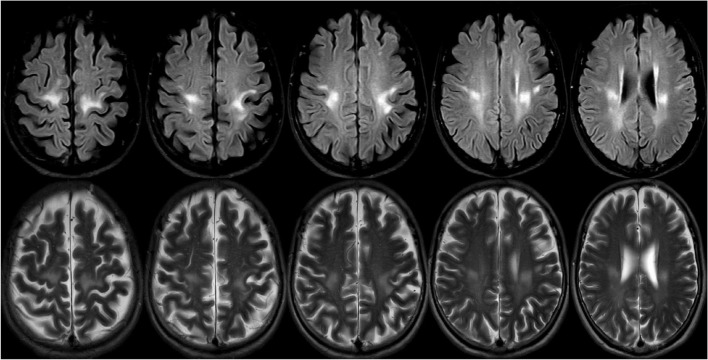
A 69-years-old female presented with a 2-year history of progressive weakness of the lower limbs. A brain magnetic resonance imaging (MRI) revealed abnormal T2 and flair hyperintensities in the periventricular and deep cerebral white matter, and the bilateral subcortical areas of parietal white matter (the lesions were continuous and symmetrically distributed). Reduced galactocerebrosidase activity and GALC gene confirmed the diagnosis of adult late-onset GLD.

Through this diagnostic process and strategy, a 67-year-old patient was diagnosed with late adult onset GLD in our clinical center. This patient presented with chronic progressive spastic gait, mild cognitive impairment, and anxiety, and there was no clinical symptom of peripheral nerve involvement. However, the electrophysiological examination results suggested decreased nerve conduction velocity and demyelination. The clinical symptoms of this patient were consistent with the typical clinical manifestations of late adult onset GLD, and this patient has been the oldest case reported in China (Figure 5).

## Conclusion

Adult-onset GLD is a rare degenerative disease of the CNS caused by abnormal lysosomal storage. This disease is characterized by chronic progressive spastic paralysis and gait disorders, with or without peripheral nerve injury, cognitive impairment, and mental disorders. Although there is no effective therapy for adult-onset GLD, this disease is expected to be eliminated by pharmacological chaperones based on animal models to improve the activity GALC enzyme, viral vectors for gene therapy, and neural progenitor cells injected into the CNS. To treat GLD, clinicians should accurately differentiate it from other acquired white matter demyelination diseases of the CNS based on the clinical and MRI features of this disease, to avoid misdiagnoses.

## Author contributions

ZL and JL contributed to the study design and drafted the manuscript. GW and XL edited the manuscript. JZ, PZ, and GL contributed to MRI of the data in the literature. MW supervised the writing of the manuscript and critically reviewed the manuscript. All authors contributed to the article and approved the submitted version.
